# Decision-Making in Management of the Complex Trauma Patient: Changing the Mindset of the non-trauma Surgeon

**DOI:** 10.1007/s00268-018-4460-x

**Published:** 2018-01-16

**Authors:** Linda Sonesson, Kenneth Boffard, Lars Lundberg, Martin Rydmark, Klas Karlgren

**Affiliations:** 1 Swedish Armed Forces Centre for Defence Medicine, Göta Älvsgatan, 426 76 Västra Frölunda, Sweden; 2 Institute of Clinical Sciences, Department of Surgery, Sahlgrenska Academy, University of Gothenburg, 413 45 Gothenburg, Sweden; 3 Department of Surgery, Milpark Hospital Academic Trauma Center, University of the Witwatersrand, Johannesburg, South Africa; 4 Department of Surgery, University of Witwatersrand, 17 Pallinghurst Road, Parktown, ZA-Johannesburg 2193 South Africa; 5 Mednet, Institute of Biomedicine, Sahlgrenska Academy, University of Gothenburg, Medicinaregatan 7 B, 405 30 Gothenburg, Sweden; 6 Department of Learning Informatics Management and Ethics, Karolinska Institutet, Tomtebodavägen 18 A, 171 77 Stockholm, Sweden

## Abstract

**Background:**

European surgeons are frequently subspecialized and trained primarily in elective surgical techniques. As trauma leaders, they may occasionally have to deal with complex polytrauma, advanced management techniques, differing priorities, and the need for multidisciplinary care. There is a lack of expertise, experience, and a low trauma volume, as well as a lack of research, with limited support as to the decision-making and teaching challenges present. We studied what experienced trauma experts describe as the challenges that are specific to the advanced surgical decision-making required, whether civilian, humanitarian, or military.

**Methods:**

Design-based research using combined methods including interviews, reviews of authentic trauma cases, and video-recorded resuscitations performed at a high-volume civilian academic trauma center.

**Results:**

Several educational dilemmas were identified: (1) thinking physiologically, (2) the application of damage control resuscitation and surgery, (3) differing priorities and time management, (4) impact of environment, (5) managing limited resources, (6) lack of general surgical skills, (7) different cultural behavior, and (8) ethical issues.

**Conclusion:**

The challenges presented, and the educational domains identified, constitute a basis for improved development of education and training in complex surgical decision-making. This study contributes new knowledge about the mindset required for decision-making in patients with complex multisystem trauma and competing priorities of care. This is, especially important in countries having a low intensity of trauma in both military and civilian environments, and consequential limited skills, and lack of expertise. Guidelines focused on the same decision-making process, using virtual patients and blended learning, can be developed.

**Electronic supplementary material:**

The online version of this article (10.1007/s00268-018-4460-x) contains supplementary material, which is available to authorized users.

## Introduction

Increasing threats with mass casualties in the civilian environment, and the rising incidence of severe multisystem polytrauma due to terror, has contributed to concern, especially in the Nordic countries and Western Europe, regarding lack of experience and expertise in the management of such trauma [1]. Surgeons have a key competence and may lead the trauma team, but those in European countries are often subspecialized, and may lack the understanding and skills necessary for management of the complex trauma patient. This requires a multifaceted approach in the hemodynamically unstable patient presenting with multiple injuries and requires the setting priorities of care, supporting a team approach, and the leadership, and expertise necessary for advanced management. These variables can impact patient survival [1]. Studies have shown that clinicians often rely on pattern recognition and heuristics to assess injuries rapidly, but an overreliance on these approaches can result in diagnostic errors [2, 3]. In stressful environments such as trauma centers, clinicians often adopt these strategies to reduce cognitive load [3, 4]. Trauma care is often characterized by uncertainty resulting in insecurity, and as such, gaps between knowledge and routine practice may result [2, 3]. In addition, a low level of evidence for many recommendations probably complicates matters further [2]. Surgeons primarily having an elective practice, may lack understanding of the multifaceted nature and management priorities in the trauma patient with competing multiple injury patterns [5, 6]. Managing these trauma patients requires the appreciation of the spectrum of injury, working under time pressure with limited equipment and resources, and integration of the decisions and the actions required [5]. This context of trauma might be extremely challenging for surgeons in environments where expertise, experience, or volumes are low. Experienced international trauma surgeons responsible for the training of those with less experience in trauma state that the mindset and the management priorities differ considerably compared to those who do have high-volume experience. In the military environment, human resources are very limited, so many civilian surgeons, anesthesiologists, and nurses work both in the civilian and austere or military medical environments. However, there is a lack of research on what the difficulties of teaching and training in the management of these patients actually are [7]. The aim was to study what international experienced trauma surgeons describe as challenges in education specific to surgical decision-making in trauma, and the mindset required, as well as training in the technical skills needed, to manage these particularly difficult trauma patients.

## Materials and methods

### Study design

**Fig. 1:**
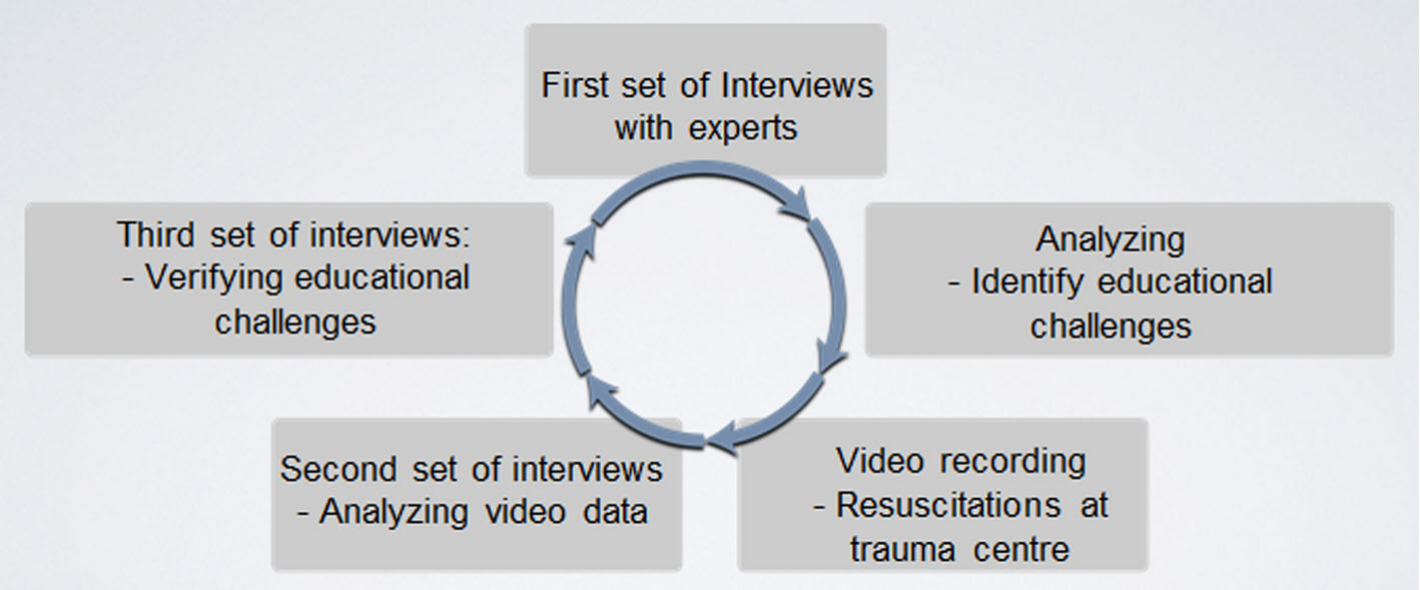
Study design with combined methods, iterative processes, and close interaction with clinicians

A design-based research approach was applied [7–9], and a close interaction between researchers and the international trauma experts was achieved. The study design consisted of combined methods such as semi-structured interviews, trauma case reviews, and video-recorded resuscitations from a major high-volume civilian academic trauma center where team training and leadership were particularly emphasized [10–16]. The study was also distinguished by iterative processes, and the findings have been verified by the researchers in interaction with the international experts [8, 9] (Fig. 1).

### The sample and setting

Eleven international experts with profound expertise in the management of complex surgical trauma, from Canada, New Zealand, Norway, South Africa, Sweden, UK, and the USA, participated individually in semi-structured interviews. Nine of these experts also supported the verification of the results. The participating experts were recruited from an international network, the International Association for Trauma Surgery and Intensive Care (IATSIC), and the senior instructor faculty of an existing course, “Definitive Surgical Trauma Care” (DSTC™). DSTC is a high-end international course offering education in the decision-making and training in the technical skills required for the management of the patient with multiple trauma injuries primarily in the civilian environment and the competing priorities that occur [5]. The military version of the DSTC course, which is an additional 1–2 days, highlights the management required of injuries from military-style weapons. The combined course was the setting of the study, since military medical resources in the Nordic countries especially are limited and health care professionals from the civilian health care sector are utilized, to provide care under austere conditions or in the military environment.

### Data and analysis

A first set of semi-structured interviews focused on the decision process in a complex trauma patient and was responded individually. A think-aloud method was used to lay bare the international experts’ reasoning in the management of civilian and military trauma cases. The authentic cases highlighted the management required in scenarios of patients with penetrating injuries, particularly gun shots, as well as those with burns [10–12]. Thematic analysis was used to analyze patterns from the data sources which then identified eight educational challenges, specific for education and training in decision-making, in the care of the complex patient with civilian or military trauma injury [15, 16]. The educational challenges were investigated through a second set of semi-structured interviews and video recordings of trauma experts and teams managing injuries due to penetrating trauma or burns, at a major civilian academic trauma center. The data source for the second set of video-recorded interviews consisted of sessions of recorded resuscitations and interviews with trauma experts in the civilian setting and aimed to investigate if the educational challenges were also the same as those identified in military clinical practice [13, 14, 16]. The same challenges and decision-making were verified by a third set of semi-structured interviews separately focusing on the specifics of teaching and training of the less experienced surgeons [8, 11, 12] (Fig. 1).

## Ethics approval and consent to participate

The study followed international guidelines for research ethics [17, 18] and participation in any interviews was optional. Information about the study, and consent to participate, was given both in writing and orally (Appendix 1 and 2, ESM). An application for ethical vetting for interviews and video-recorded observations of management of actual trauma cases at a major academic trauma center was approved by the Swedish National Board of Ethics and Human Research Ethics Committee (Medical) of the University of the Witwatersrand, Johannesburg, South Africa (Appendix 3 and 4, ESM).

## Results

Eight educational challenges were found to represent domains including those aspects which were particularly difficult to master, but which are required for decision-making in management of the complex trauma patient. These domains were identified from different sources of data and verified by the trauma experts. The challenges were ranked by the trauma experts and start with the most important challenge for the expert to teach and to stimulate the broader mindset, required for the course participant. An overview is presented in Table 1 and commented upon in detail below.

**Table 1:** Educational challenges in advanced civilian and military trauma care verified by nine international trauma experts (E1–E9)

Educational challenge	E1	E2	E3	E4	E5	E6	E7	E8
1. Thinking physiologically	X	X	X	X	X	X	X	X
2. Damage control surgery	X	X	X	X	X	X	X	X
3. Priorities and time management	X	X	X	X	X	X	X	X
4. Impact of environment	X	X	X	X	X	X	X	X
5. Managing limited resources	X	X	X	(X)	(X)	X	X	X
6. Lack of general surgical skills	X	X	X	X	X	X	X	X
7. Different cultural behavior	X	X	X	(X)	–	–	–	–
8. Ethical issues	X	–	X	–	–	–	–	X

X stated as an educational challenge, (X) depends on the setting, – no educational challenge

### Thinking physiologically

This was ranked as the most important of the identified educational challenges when it comes to both teaching and learning advanced trauma management. The physiology (not the actual injury) determines what should be managed and prioritized first and was therefore seen as most important to learn. An understanding of the physiological changes resulting is the prerequisite for management of the unstable patient. The pitfalls for non-trauma surgeons were related to the lack of knowledge and experience about the nature of multiple system trauma and the associated physiological derangements, resulting in a flawed decision-making process.

### Damage control resuscitation and surgery

For the experts, stimulating participants to fully understand the principles of damage control resuscitation and surgery required that their reasoning into broader physiological perspectives be broadened. This was considered as challenging and important—interpreting surgical trauma damage and knowing what specific actions were required. This challenge is linked to the previous one, thinking physiologically, and represents the surgical solution to a physiological problem, using specific approaches and techniques.

### Priorities and time management

Different interpretations of the priority of care in the understanding of “when to wait, and when not to wait” was seen as a fundamental educational challenge related to the mindset in care of such patients. Patients with multiple injuries may be hemodynamically unstable, and time for diagnosis is limited. The challenge for participants is to start treating the patient without knowing precisely what is wrong, perhaps without a full diagnosis, and without the confidence and the knowledge to use the limited time well. According to the experts, course participants, unfamiliar with the priorities and decisions required, tended to prolong the management of the complex trauma patient, which resulted in loss of valuable time needed to save the life of the patient.

### Impact of environment

The extremes of austere or military medical environments are hard to visualize, and therefore difficult for participants to relate to. The challenge for less experienced surgeons seems to be twofold: to work during extreme conditions and to understand the impact of an extreme environment on the physiology of the trauma patient.

### Managing limited resources

A challenge for the mindset in relation to decision-making and during time pressure often occurred when there were limited resources in management of the patient. Less experienced surgeons are used to the situation at their civilian hospitals, with some redundancy regarding personnel and medical equipment. In a military or austere medical setting, these resources are limited, and the experts maintained that less experienced surgeons had difficulties in learning how to adapt to such situations.

### Lack of general surgical skills

The trauma experts highlighted the development of enhanced noninvasive and subspecialty skills at the expense of maintaining the general surgical open skills required for management of advanced trauma cases as a major challenge. The lack of equipment and attrition of general surgical skills becomes most challenging in the austere or military medical environment. This puts a great demand on the non-trauma surgeon to be able to apply these general surgical skills with confidence.

### Different cultural behavior

Different cultural behaviors could be challenging for the experts, when performing education and training in different countries, but the most substantial challenge was to be able to handle subcultures in multidisciplinary groups. The participants in the DSTC military version course were multidisciplinary with different professional backgrounds (e.g., anesthesiologists, endoscopic surgeons, specialized surgery nurses, and orthopedics). Additional focus of education and training was on the surgeons with practical training in team management. It was challenging for the professionals other than surgeons to actively participate in the course. Most surgeons attending the course were subspecialized in areas other than trauma, which contributed to a wider surgical knowledge within the group as well as different subcultures, and competing priorities within the group.

### Ethical issues

When taking care of trauma patients, it was stated as highly important to always do the best for the most, including whether that is to treat or to palliate. Such ethical issues may occur, e.g., during prioritizing and treatment of mass casualties. A challenge expressed by the experts was that participants need to deal with ethical issues beyond those to which they are accustomed to in usual healthcare.

## Discussion

The eight identified and verified educational challenges in the environment required for decision-making in the management of the complex trauma patient presented in the results represent particularly difficult aspects to teach and master. Not only did the international trauma experts highlight the educational challenges regarding crucial surgical skills and managing with limited resources in demanding environments, they primarily described the challenge of learning advanced trauma, as a matter of acquiring a certain mindset specific for management of that patient. This requires a radically different way of thinking and making decisions compared to what the health professionals are used to doing in their usual healthcare environments, and within the subspecialties in which they normally operate.

Established trauma surgeons like the international experts have specialized expertise in management of the complex trauma case, as well as research experience in the field of trauma. Therefore, they have the knowledge, confidence, and experience to fall back on when time and resources are limited. Finally, existing trauma guidelines, medical ethics, and advanced research in trauma are generally focused on management of the injuries themselves, but do not adequately capture this complexity in the presence of competing priorities of care. This needs to be addressed in research as well as in more focused education and training.

The presented eight educational challenges constitute domains and are a basis for focused improvement in the development of education and training techniques in trauma surgical decision-making. The result from this study also contributes to new knowledge about the characteristics of the special mindset required for the management in major trauma, especially where that knowledge is lacking or forgotten in countries with a limited expertise, experience or volumes of trauma. The eight identified domains will be developed into guidelines aimed at improving future solutions. The guidelines consist of specific design concepts based on previous studies [7] and include digital components such as virtual patients (VP) which might support and stimulate decision-making in both civilian and military trauma. VPs that are taken from actual patient scenarios as used on the DSTC course are authentic, and the progression of the case and eventual outcome can be varied electronically depending on the management choices made, including the provision for negative outcomes. VPs might contribute to stimulation of the mindset and reasoning ability among course participants and support collaborative learning in team training but also as support for decision-making in clinical practice.

This educational design concept supports blended learning, contributes to better time management, and supports a more flexible form of learning, through the use of digital technology in combination with physical parts of a course. Blended learning provides contributions of learning and training regardless of time and can be used before, during, or after a course.

Applying design-based research and its characteristics of iterative processes and close collaboration with the international experts has contributed with strength and validity of the study design [7–9]. By using combined methods like several sets of interviews and video-recorded observations, this has also contributed to an extended data from different sources [10, 13, 14]. The verification of the results, in collaboration with the international experts, has made it possible to provide a deeper understanding of the underlying causes of the challenges in education in multisystem trauma, and the competing priorities of care [12, 15, 16]. The combined methods in the study and the close collaboration with the international experts contributed to the reliability of the results in the study [9, 11].

## Conclusion

The eight educational challenges presented in the results constitute domains and are the basis for improvement and development of education and training in complex surgical decision-making. This study contributes to new knowledge about the mindset required for decision-making in multisystem trauma, where competing priorities are present and skills are limited, in countries with lack of expertise, experience, and low intensity of civilian and military trauma. The educational domains will be developed into a design concept, including digital components such as virtual patients (VP) which support and stimulate decision-making in trauma. VPs can stimulate a change in the mindset and reasoning ability among course participants and collaborative learning in team training and support decision-making in clinical practice. This educational design concept supports the development of how to blend education and training into blended learning.

## Electronic supplementary material

Below is the link to the electronic supplementary material.

Supplementary material 1 (PDF 155 kb)

Supplementary material 2 (PDF 315 kb)

Supplementary material 3 (PDF 331 kb)

Supplementary material 4 (PDF 346 kb)
