# Supplementary material for: Decision-Making in Management of the Complex Trauma Patient: Changing the Mindset of the non-trauma Surgeon
Source: World J Surg. 2018 Jan 16;42(8):2392–7. doi: 10.1007/s00268-018-4460-x (PMC6060797; doi:10.1007/s00268-018-4460-x)
Supplement: Supplementary file 1 — Supplementary material 1 (PDF 155 kb) [file 268_2018_4460_MOESM1_ESM.pdf]

Department of Learning, Informatics, Management and Ethics (LIME)
PhD student
Linda Sonesson, RN, MSc
Mail: linda.sonesson@ki.se
Mobile: + 46 (0)721-877459

To:
Experts in Advanced civilian and/or military Trauma

Interview – going through Cases

Aim of study
The aim with interviews is to identify "the right way" using decision points through each case. Data from the interviews will be used to analyze the pedagogical challenges, identify and describe design principles to be able to developing eLearning support (a "Virtual Patient") for physicians and nurses within advanced civil and military trauma. The eLearning support shall be able to be used both during physical lectures (discussions), and before or after a course.

The data collection is a part of sub study 4 in the PhD project: *Pedagogical Design and Educational Challenges in Advanced Civilian and Military Trauma Care*

About the interview
The interview will be video recorded while going through case: 23 and 01 from DSTC, and takes about an hour.
The purpose with video recordings in this sub study is to visualize and document answers to the questions, as well as the discussion that results. Those interviewed, have the right to see the material of themselves if they wish to do so, and also the right to deny the use of data. If so the data will be destroyed. All data will be saved on external media and locked. Supervisors and PhD student will have the right to all data and the data will not be used for any other purpose than as documentation.

Questions
While going through each case the questions will be:

1. How would you like to solve the Case? (think-aloud and step-by step)
2. What are the educational/teaching challenges in this case as you see it?
3. Which are the key decision points in this case as you see it?
4. What are the challenges (for the participant) in relation to the choice at each decision point?
5. What is the critical in the decision that the participant has to make?
6. When/where in the case does a participant often make the wrong decision?
7. How should the participant be guided towards the best decision path?
