# Supplementary material for: Decision-Making in Management of the Complex Trauma Patient: Changing the Mindset of the non-trauma Surgeon
Source: World J Surg. 2018 Jan 16;42(8):2392–7. doi: 10.1007/s00268-018-4460-x (PMC6060797; doi:10.1007/s00268-018-4460-x)
Supplement: Supplementary file 2 — Supplementary material 2 (PDF 315 kb) [file 268_2018_4460_MOESM2_ESM.pdf]

A Satellite Training Centre of the
University of the Witwatersrand
Johannesburg

Department of Trauma Surgery: Netcare Milpark Hospital

P. O. Box 91155, Auckland Park, 2006, South Africa • Telephone and Fax +27 (0)11 482-4889 • Email: mptrauma@mweb.co.za

Consent to participate in a research project about education and training in advanced civilian and military trauma

Good day, I am Prof KD Boffard Professor Emeritus, Department of Surgery, University of the Witwatersrand and Trauma Director and Academic Head, Milpark Hospital Academic Trauma Centre, University of the Witwatersrand

Project Title:
"Digital educational models that support advanced civilian and military traumatology"

Invitation:
We are inviting you to take part in a research study and requesting for your permission to participation and to use material videotaped during a resuscitation event. For a period about one week in the end of February 2017, a research team will conduct a study at Milpark Hospital Academic Trauma Centre. In order to proceed with this project, consent is needed from you as a professional in the trauma team.

The aim of study and expected outcomes:
The aim is to study experienced trauma surgeons reasoning and decision-making of cases in complex surgical trauma through video recorded observations and debriefing interviews.
The data will be used to analyze the educational challenges in education and training of surgeons, anesthesiologists and scrub nurses within complex surgical trauma.

Expected outcomes from the study are identified design principles, needed for the development of digital support for education and training within advanced civil and military trauma for physicians and nurses.

Information about research project
The project forms part of a joint project which will take place between the Department of Learning Informatics Management and Ethics (LIME) at Karolinska Institute, Stockholm, and the Department of Surgery of the University of the Witwatersrand, through the Department of Surgery, and the Milpark Hospital Trauma Centre, both of which are approved training centres of the University of the Witwatersrand Department of Surgery, and the Health Professions Council of South Africa.
The Senior Investigator in the study, Klas Karlgren, senior researcher at the Department of LIME, Karolinska Institute, and the Co-Investigator, Ms. Linda Sonesson have extensive experience in research methods and ethical issues related to the use of video recording in medical education and research.

Financial
The project is wholly supported by Department of LIME the Karolinska Institute, and the Centre for Defence Medicine, Swedish Armed Forces. There is no personal financial benefit to any member of the participating group, nor any participating organisation, other than the covering of any direct expenses incurred.

Background to the study:
The project aims to contribute to knowledge of the specific educational challenges that are found in advanced traumatology and military medicine and to study the difficulties that may be met in the particularly demanding cases, as well as how technology supported learning can be developed and

utilized to prepare and train the target audience for the work in advanced traumatology and defence medicine both nationally and internationally.
This knowledge gain can be of great importance for the training of doctors and nurses to be trained in the area. Since doctors and nurses in the Nordic countries encounter severe trauma cases far less often than their equivalents in South Africa, there are great opportunities to use technology supported learning and virtual patients for preparation and training. With more and better preparation, programs of education quality can be improved, which ultimately could save lives especially in more extreme situations, both civilian and military. Examples include patients with bullet and explosion damage, in areas where resource challenges are present – either physical resources, are when the patient load exceeds the ability to deal with the situation.

The research method

Video recorded observations and debriefing interviews

The purpose of using video recorded observations and debriefing interviews of advanced trauma at Trauma Centre is to be able to study experienced trauma surgeons reasoning and decision-making with the team. The aim is to identify design principles for developing digital support for education and training. The method includes two parts of video recording: *1. Observations of resuscitations* and *2. Debriefing interviews*. The focus in the study is not the patient or individuals, the focus are reasoning in decision-making and interaction in the team.

Video recordings are going to be used as a method for documentation and analysis of applied decision-making in resuscitations. Video recording as a method also provides the possibility to iteratively analyze and understand different aspects of decision-making together with the research participants. The analysis of the video recordings can clarify the practices that unfold and have a focus on the behaviors and interactions between the participants including talk, pauses, turn-taking, gestures, movement, postures, facial expressions and the use of various artifacts (equipment, documentation, tools). Video data serve an important role in supporting observation, documentation and analysis as complex and volatile as teamwork in advanced trauma settings. Video data can therefore contribute to a better understanding of decision-making and teamwork which is hard or impossible to study by using other research methods; the details of the teamwork in a trauma care team would not be possible to relate in a retrospective interview or questionnaire.

Study protocol – Observations and team interviews

1. *Observations of trauma resuscitations*

During trauma resuscitation, the resuscitation room and its staff interactions will be video recorded by a member of the team. The focus is on staff interaction in the team and <u>not</u> on the patient.
By using a think-aloud method during video recording trauma resuscitations the trauma leader is asked to reasoning aloud during the resuscitation (while treating the patient):

Questions for the trauma leader (repeated step-by-step during the treatment)

- What has happened?
- What resources do you have (equipment, humans)?
- What will you prioritize?

2. *Debriefing interviews with the trauma team after resuscitation*

After the resuscitation is complete, and the patient has left the area, the team will be interviewed in association with the video in the form of a debriefing. Their input will be included as to how the resuscitation went and whether there were any areas for improvement. Going through the video recorded resuscitation together with the team leader and the trauma team using debriefing questions as:
- What are you satisfied with/what did you do well?
- What did the team did well?
- Which were the critical decision points in treatment of the patient as you see it?

- Which were the challenges in relation to your choices and the decision points?
- What could you improve by yourself until next time?
- What does the team need to improve?
- How would you like to solve the Case? (think-aloud and step-by step)

Consent to participate
The research project has received ethical approval from the regional ethical review board in Stockholm, Sweden (appendix 1) and all work will also be done in accordance with the European law of Personal Data Act (1998:204) and the *Declaration of Helsinki – Ethical Principles for Medical Research Including Human Subjects* (WMA, 2013). A research participant defines here as staff and the patient in the resuscitation room. All research participants who are part of the recordings will be anonymous in the reports from the project. When it comes to trauma patients consent in research studies European ethical guidelines suggests local ethical approval for each trauma unit (WMA, 2013). Research participants will be protected anonymous by using technical technique which aloud blurring of individual faces and changing voices.
Research participants will have the right at any time cancel their participation. If participation is canceled the material including the specific research participant will not to be used.
The video recordings will not be used for other purposes than documentation and analysis and the data will not be shared or visualized. Recordings will be stored at external hard drives and locked up in such a way that no unauthorized persons can gain access to the data. Only the research project group will have access to the collected material.

Benefits:
Benefits that can occur is that the more people learn more about traumatology and by digital training environments evolve, it may mean a reduced need for training on real patients, and a better quality of care.

Risks:
There are no risks predicted.

Consent and confidentiality
During your participation in interviews and observations you will be informed orally and in writing of the doctoral research project, and have the right to cancel your participation and involvement at any time should you feel uncomfortable for any reason. No questions will be asked, and no reason need to be given for withdrawal. All data will be treated with confidentiality. Participation in the study will not affect your participation in any courses and you may choose at any time to cancel without having to state the reason and without incurring any consequences for the participation in the course.

You are invited to take part in the data collection relating to this participation and access results and data information about how the data is managed and how long.

Data will de-identified before the results are published or made public.

Contact details of researcher:
For further information or questions please contact:
Prof KD Boffard on 0825514960
Or
Dr R Jacks on 0829047554

Contact details of REC administrator and chair:

If you have any questions about your rights as a research participant, or you would like to obtain information or offer input, or you wish to speak with someone **not** directly involved with the study, you should contact:

Prof. Cleaton-Jones, Chairperson of the University of the Witwatersrand, Human Research Ethics Committee (HREC), which is an independent committee established to help protect the rights of research participants at (011) 717 2301.

Thank you for your participation and we look forward to working with you!
You are free to withdraw at any stage simply by letting us know. Sign the consent form and let us know if you are willing to participate or not.

□ Yes, I want to participate. The video recordings can only be used in research of education and training for advanced civilian and military trauma.
□ No, I do not want to participate in the study.

Participant Signature: ______________________ Date: ________/_______/_____

Full Name and Surname: ____

Investigator Signature: ______________________ Date: ________/______ /_____

Full Name and Surname: ____
