# Supplementary material for: Decision-Making in Management of the Complex Trauma Patient: Changing the Mindset of the non-trauma Surgeon
Source: World J Surg. 2018 Jan 16;42(8):2392–7. doi: 10.1007/s00268-018-4460-x (PMC6060797; doi:10.1007/s00268-018-4460-x)
Supplement: Supplementary file 3 — Supplementary material 3 (PDF 331 kb) [file 268_2018_4460_MOESM3_ESM.pdf]

R14/49 Prof Kenneth David Boffard et al

HUMAN RESEARCH ETHICS COMMITTEE (MEDICAL)

CLEARANCE CERTIFICATE NO. M161113

NAME: (Principal Investigator) Prof Kenneth David Boffard et al

DEPARTMENT: Surgery
Netcare Milpark Academic (TSSA Level I) Trauma Centre

PROJECT TITLE: Digital Education Models that Support Advanced Civilian and Military Traumatology

DATE CONSIDERED: 25/11/2016

DECISION: Approved unconditionally

CONDITIONS:

SUPERVISOR:

APPROVED BY:

Professor P Cleaton-Jones,Chairperson, HREC (Medical)

DATE OF APPROVAL: 11/01/2017

This clearance certificate is valid for 5 years from date of approval. Extension may be applied for.

DECLARATION OF INVESTIGATORS

To be completed in duplicate and **ONE COPY** returned to the Research Office Secretary in Room 301, Third Floor, Faculty of Health Sciences, Phillip Tobias Building, 29 Princess of Wales Terrace, Parktown, 2193, University of the Witwatersrand. I/we fully understand the conditions under which I am/we are authorized to carry out the above-mentioned research and I/we undertake to ensure compliance with these conditions. Should any departure be contemplated, from the research protocol as approved, I/we undertake to resubmit the application to the Committee. **I agree to submit a yearly progress report**. The date for annual re-certification will be one year after the date of convened meeting where the study was initially reviewed. In this case, the study was initially reviewed in November and will therefore be due in the month of November each year. Unreported changes to the application may invalidate the clearance given by the HREC (Medical).

Principal Investigator Signature

Date

PLEASE QUOTE THE PROTOCOL NUMBER IN ALL ENQUIRIES
