# Supplementary material for: Decision-Making in Management of the Complex Trauma Patient: Changing the Mindset of the non-trauma Surgeon
Source: World J Surg. 2018 Jan 16;42(8):2392–7. doi: 10.1007/s00268-018-4460-x (PMC6060797; doi:10.1007/s00268-018-4460-x)
Supplement: Supplementary file 4 — Supplementary material 4 (PDF 346 kb) [file 268_2018_4460_MOESM4_ESM.pdf]

Translation of the decision from the Regional Ethical Review Board of Stockholm with reference number: 2016/1701-31/5

The decision ("beslut"), explanation ("motivering") and advisory statement ("rådgivande yttrande") (cf. page 2) from the Ethics Board with reference number 2016/1701-31/5 have been translated into English:

Applicant: Swedish Armed Forces
Qualified representative: Anders Sjöholm
Project: Digital educational models as support for advanced civilian and military traumatology
Researcher (responsible for) conducting the research: Klas Karlgren

----

Tomas Månsson announces conflict of interest and leaves the meeting room.

Decision
The board will not process the application for ethical approval

Explanation
The research that the applicant intends to carry out does not involve handling of personal information as specified in § 3 of the Ethical Review Act. The research is thus not encompassed by the Ethical Review Act. The application for approval of the research is therefore not processed.

According to statute 4a § (2003:615) of the Ethical Review of Research Involving Humans, the board leaves the following

Advisory statement
The board does not have any ethical objections against the study under condition that the information letter is supplemented with information about who is responsible for personal data (organization/responsible research body) as well as other information needed for registered participants to take charge of their rights in connection to the treatment such as information about the right to apply for information (printout from the dataset) and to get corrections of possibly incorrect data.

How to appeal the decision about not processing this application for acceptance: The advisory statement cannot be appealed.

Attest of correspondence to the original source text

Italo Masiello

Head of Clinical Skills and Innovation Centre at the South General Hospital and Associate Professor at Karolinska Institutet

Stockholm, 2016-12-27
